# Progress and prospects of organoids in the pathogenesis of lung cancer and screening of antitumor drugs

**DOI:** 10.3389/fonc.2025.1734929

**Published:** 2026-01-14

**Authors:** Yiqiong Li, Yuanjie Shang, Na Zhang, Jiarui Li, Zijiang Yang

**Affiliations:** Medical College, Henan University of Chinese Medicine, Zhengzhou, China

**Keywords:** antitumor drug screens, lung cancer, organoids, pathogenesis, precision medicine

## Abstract

Lung cancer, a major global public health issue for decades, is characterized by high incidence and mortality rates. Over the past twenty years, numerous novel therapies have emerged. However, due to the substantial genetic and phenotypic heterogeneity among patients, current treatment modalities still yield low cure rates for advanced lung cancer, creating an urgent need to develop precision medicine aided by personalized tumor models. The rise and advancement of three-dimensional organoid culture technology have brought new hope to this endeavor. Patient-derived organoids can closely mimic the *in vivo* biological characteristics of lung cancer, enabling the exploration of its pathogenesis and the effective screening of anticancer drugs, thereby facilitating progress in precision medicine for lung cancer. This review provides a systematic analysis by constructing a framework that spans foundational techniques, mechanistic investigations, preclinical applications, and integration with emerging technologies. It specifically highlights the role of organoids in simulating the dynamic evolution of lung cancer, deciphering tumor heterogeneity, and serving as a versatile platform for convergence with cutting-edge tools such as organs-on-chips, 3D bioprinting, and CRISPR-Cas9. Furthermore, the review discusses the key challenges and limitations facing lung cancer organoid models and outlines their future prospects for broader application.

## 1 Introduction

With the increase in global population and the intensification of aging trends, cancer has become the leading cause of premature death and reduced life expectancy in many countries. Lung cancer, a malignant tumor originating from respiratory epithelial cells (bronchi, bronchioles, and alveoli), ranks first globally in terms of incidence rate and mortality rate due to its complex pathogenic mechanisms, long latent phase, and difficulty in early detection (1). According to data released by the WHO (GLOBOCAN 2022) (2), there were approximately 20 million new cases of cancer globally, with about 9. 7 million deaths. Among these, nearly 2. 48 million were new cases of lung cancer (12. 4%), and about 1. 8 million were deaths (18. 7%), making it the most frequently diagnosed cancer. Furthermore, as the Human Development Index (HDI) increases, the incidence and mortality rates of lung cancer have also been rising year by year (3). Lung cancer is primarily divided into two major categories: non-small cell lung cancer (NSCLC) and small cell lung cancer (SCLC) (4). Non-small cell lung cancer is the most common pathological type, accounting for approximately 80% to 85% of all lung cancers (4). The development and progression of lung cancer is a multistage, multigenic process. At its core lies the accumulation of genetic and epigenetic alterations that ultimately disrupt key regulatory mechanisms governing cell proliferation, differentiation, and apoptosis, thereby inducing carcinogenesis. Currently, epidermal growth factor receptor (*EGFR*) mutations, anaplastic lymphoma kinase (*ALK*) rearrangements, and *Kirsten rat sarcoma virus* (*KRAS*) mutations have been identified as key oncogenic drivers. Overexpression of genes such as *HER*, *MYC*, *Sox*, *MDM2*, and *ERBB2*, along with the loss or mutation of *TP53*, *RB1*, *CDKN2A*, *NME1*, and *PTEN* have also been demonstrated to be closely associated with lung cancer development (5–7). Other relevant molecular mechanisms include activation of growth factor signaling pathways (e. g., PI3K/AKT/mTOR, RAS/RAF/MEK/ERK), tumor angiogenesis, impaired apoptosis, and immune evasion. In recent years, with technological innovations and advancements in medical care, research on precision diagnosis and treatment of lung cancer has been abundant and continuously deepening. Traditional treatment methods such as surgery, radiotherapy, and drug therapy have gradually matured, and significant breakthroughs have been made in neoadjuvant immunotherapy. Targeted therapy based on histological characteristics and biomarker expression levels has developed rapidly (8). However, due to various special properties of tumor cells, such as genetic instability, recurrence and metastasis, and individual differences (9), precision medicine based on genomics cannot accurately address the unpredictable nature of tumor cells due to its inherent limitations, resulting in persistently high incidence and mortality rates of lung cancer. With the increasing maturity of organoid technology, it has become possible to establish patient-derived lung cancer organoids (PDLCOs). The emergence of this precision model not only offers new insights into tumor biology and disease progression but also provides robust support for identifying novel therapeutic targets and biomarkers.

Organoids primarily refer to *in vitro* models established through three-dimensional culture of adult stem cells (ASC), induced pluripotent stem cells (iPSC), or embryonic stem cells (ESC), which can highly simulate the structure and function of the original tissue or organ (10). Lung cancer organoids (LCOs) provide a crucial platform for studying lung cancer heterogeneity. However, whether their cellular composition accurately reflects the proportion of cellular states in the primary tumor remains a question warranting further investigation. It is important to clarify that organoids are not equivalent to simple aggregates of stem cells; ideally, they should contain cells at various stages of differentiation (**Table 1**). However, during standardized establishment, culture conditions selectively favor the growth of cell subpopulations with stem cell characteristics or strong proliferative capacity, while those with slow proliferation or terminal differentiation are prone to loss (11). Consequently, organoids obtained through routine culture more accurately reflect the amplifiable potential of tumor cells rather than their *in situ* proportions. Optimizing culture protocols, such as adjusting growth factor combinations or employing more biomimetic matrices, can partially enhance the heterogeneity retained in the culture system. On the other hand, even if primary tumor maintenance does not strictly depend on preexisting stem cell populations, organoid culture systems may induce stem-like characteristics by activating cancer cell phenotypic plasticity and triggering dedifferentiation in non-stem cells (12, 13). Despite these complex mechanisms, organoid models derived from patients still exhibit numerous unique advantages over traditional research models. They can largely preserve the genetic background, tissue architecture, and functional characteristics of the target tissue or organ, simulate the process of tumor cell plasticity transformation, and open new avenues for in-depth exploration of lung cancer development and metastasis mechanisms, as well as rapid and efficient screening of anti-tumor drugs. They hold tremendous potential for achieving personalized treatment for lung cancer patients. This article reviews the development history, technical basis, application in lung cancer research, integration with emerging technologies, and future application prospects of organoid technology. The aim is to provide theoretical guidance for translating LCO-related research findings into clinical practice and to promote the development of precision medicine for lung cancer. Precision medicine is an emerging approach to disease treatment and prevention that emphasizes developing personalized diagnostic and therapeutic strategies based on an individual’s unique genetic, environmental, and lifestyle information.

**Table 1:** Differences and connections between organoids and stem cells.

Characteristics	Stem cells	Organoids
Essence	A type of multipotent cell with self-renewal capacity	A three-dimensional cell culture resembling natural organs
Source	Early embryos, adult tissues, induced pluripotent stem cells	Embryonic stem cells, adult stem cells, tumor tissue cells, organ-specific progenitor cells
Composition	Nucleus, cytoplasm, cell membrane	Stem cells, extracellular matrix, blood vessels, nerves
Structure	Single-cell level, no specific macroscopic structure	Possesses a three-dimensional spatial structure, incorporating multiple cell types and partial organ-specific functions
Function	Self-renewal and multidirectional differentiation	Simulate the physiological and pathological functions of source tissues
Connection	The starting cells for organoid construction	Products and Application Forms of Stem Cells in *In Vitro* Culture
Similarities	Both exhibit self-renewal and differentiation potential, and both depend on microenvironmental signals	

## 2 Technical basis of LCOs

### 2.1 Development history of organoids

**Figure 1:**
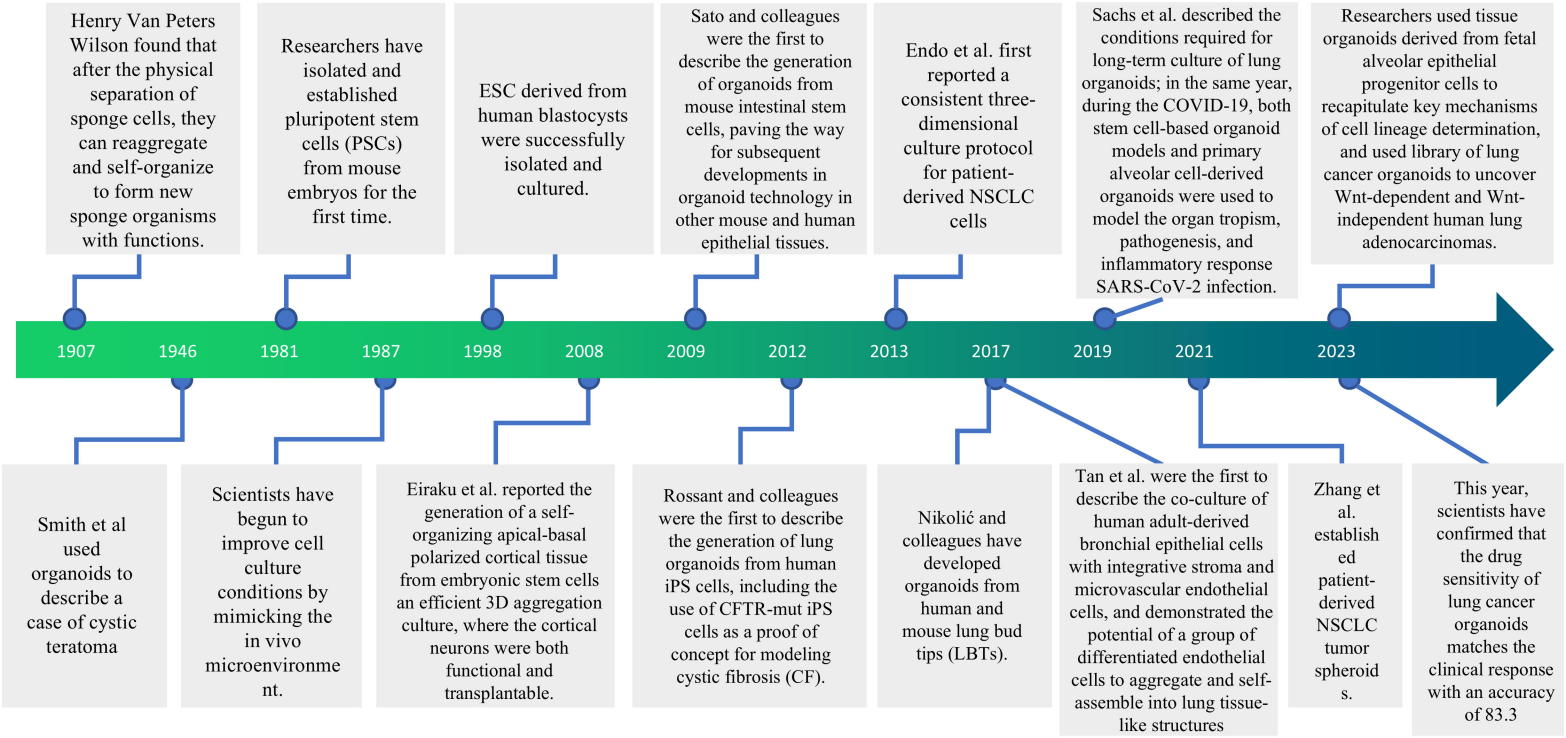
Key events in the history of organoid research.

As early as 1907, Professor Henry Van Peters Wilson of Baker College in the United States observed that sponge cells could regroup and self-organize to form a functionally normal sponge organism after physical separation, laying the initial theoretical foundation for the concept of organoids. In 1981, researchers first isolated and established pluripotent stem cells (PSC) from mouse embryos; in 1987, scientists began to focus on optimizing cell culture conditions by simulating the microenvironment *in vivo*; in 1998, ESC derived from human blastocysts were also successfully isolated and cultured. In 2008, Eiraku et al. utilized 3D aggregation culture to generate cortical tissue from ESC, marking the transition of organoid research from 2D to 3D (14). The rise of stem cell technology, tissue engineering technology, and three-dimensional culture technology has further propelled organoid research to new heights and provided solid technical support for the establishment of organoid models. In 2009, a landmark study in Hans Clevers’ laboratory—using a single mouse LGR5+ intestinal stem cell to self-organize into an intestinal crypt-villus structure *in vitro*—marked the official entry of organoid technology into a new era (15). In 2013, Dekkers et al. successfully cultured the first intestinal organoid derived from a human cystic fibrosis patient carrying a *CFTR* gene mutation, thus realizing the modeling of human diseases using organoids (16). Today, organoids have been widely applied in research across multiple human systems. In the digestive system, intestinal organoids have become crucial models for studying epithelial biology, infection, inflammatory bowel disease, and colon cancer mechanisms (15, 17); In the nervous system, brain organoids are employed to simulate neurodevelopment, autism spectrum disorders, and glioblastoma, among others (18, 19); in the reproductive system, prostate cancer organoids and ovarian cancer organoids provide powerful tools for delving into tumor heterogeneity and drug response (20, 21). Concurrently, the emergence of respiratory system organoids has created opportunities to simulate the mechanisms of lung cancer development and drug resistance (**Figure 1**).

### 2.2 Establishment of LCOs

Organoids are organ-specific three-dimensional cell culture products obtained by seeding stem cells onto a matrix gel or basement membrane extract and culturing them under the combined action of key growth factors and signaling molecules such as epidermal growth factor (EGF), noggin (NOG), and R-spondin1 (RSPO1) (22). The formation and continuation of such cultures rely on an active stem cell population and its derived cells, which can continuously expand their cell population over time. The maintenance and stable growth of their three-dimensional structure are influenced by external environmental regulation of cell behavior (23). Cells can be sourced from various origins, including standard cell lines, single-cell suspensions obtained through enzymatic digestion of tissues from needle biopsies or surgical resections, and cancer cells extracted from the blood or metastatic pleural effusion of cancer patients (24, 25). The establishment of the three-dimensional structure of cells involves various methods such as embedding growth of cells in a matrix scaffold, culturing in fixed droplets of medium utilizing gravity and surface tension, and polarization and differentiation at the air-liquid interface (26). Furthermore, three-dimensional matrices and cytokines are also essential materials required for the growth of organoids. Their combined action makes the culture system more similar to the *in vivo* microenvironment, thereby ensuring that organoids cultured *in vitro* can retain the basic characteristics of the source tissue relatively intact (27).

The successful establishment of LCOs primarily relies on several key steps, namely specimen collection, tissue preservation and transportation, tissue digestion and tumor cell isolation, organoid 3D culture and expansion, organoid passage, and drug testing. Up to now, the sources of tissue specimens for LCO culture have become very extensive, including needle biopsy specimens, surgical resection specimens, peripheral blood circulating tumor cells, pulmonary venous blood circulating tumor cells, pleural fluid, or bronchoalveolar lavage fluid. Different sampling methods can have a certain impact on the success rate of modeling. Surgical resection specimens demonstrate the highest success rate in constructing LCOs due to their significant advantages of containing diverse tissue types, sufficient cell numbers, and effectively retaining the original characteristics of tumors. In contrast, needle biopsy specimens, although simple to operate, yield limited tissue amounts. The number of peripheral blood circulating tumor cells is scarce and difficult to capture. Pulmonary venous blood circulating tumor cells have high biological activity but require high standards for sample collection. Specimens derived from pleural fluid or bronchoalveolar lavage fluid are prone to changes due to the influence of the intrathoracic microenvironment, resulting in a generally lower success rate in modeling. Following sample collection, isolated tumor cells were suspended in matrix membrane extracts (e. g., Matrigel) and cultured in specialized serum-free media supplemented with growth factors essential for maintaining epithelial stem cells (e. g., EGF, NOG, RSPO1). Under these optimized conditions, cells self-organize into three-dimensional organoid structures within 1–2 weeks. Established organoids can be validated prior to expansion using hematoxylin and eosin (H&E) staining and immunofluorescence analysis (IF). For subsequent clinically relevant experiments, such as high-throughput drug screening, organoids are typically used at early passages (e. g., passages 3-5) to ensure genetic stability and phenotypic consistency with the original tumor (28–30). In addition to the traditional immersed Matrigel culture method, gas-liquid interactive organoid culture methods and organoid chips based on microfluidic technology have gradually emerged for the 3D culture, expansion, and passage of LCOs (31). These emerging culture techniques effectively address issues such as unstable cell density control and lack of physical flow conditions in traditional organoid culture methods, greatly improving the consistency and stability of LCOs and significantly enhancing the success rate of modeling.

In terms of culture medium, basic medium, growth factors, and extracellular matrix mimics are essential components. Among them, basic medium is the core condition for organoid culture, primarily consisting of DMEM, RPMI-1640, and MEM, playing a crucial role in providing nutrients such as carbon sources, nitrogen sources, minerals, and water to cells. Various growth factors added to basic medium typically include the following four categories: Wnt signaling pathway activators such as Wnt 3a and R-spondin promote stem cell growth and proliferation; tyrosine kinase ligands such as EGF stimulate epithelial cell proliferation; TGF-β signaling pathway inhibitors such as Noggin inhibit epithelial cell differentiation; and small molecule inhibitors such as CHIR99021 and Y-27632 maintain stem cell pluripotency (32). Matrigel, extracted from mouse sarcoma, is the most common matrix material. However, there are compositional differences between different batches of Matrigel, leading to low reproducibility of culture results (33). Therefore, hydrogel systems prepared from various natural or synthetic materials have been developed and successfully applied (33). New materials such as composite hydrogels allow LCOs to be co-cultured with stromal cells and have controllable biochemical signals and independent changes in mechanical properties, making the growth, development, and morphology of LCO more controllable (34). Recently, the concept of “assemblages” has been proposed, which Pasca et al. define as self-organizing cellular systems composed of different organoids or cell types. This 3D cell co-culture system constructs a complex heterogeneous tissue microenvironment by synchronizing or stepwise mixing multiple cell types, effectively overcoming the limitations of existing LCOs and providing a powerful model for basic research and clinical application of lung cancer. It is worthy of further exploration and full development and utilization by researchers (35).

It is noteworthy that although multiple studies have reported modeling success rates for specimens from different sources (**Table 2**) (36–41), significant heterogeneity exists among these data, reflecting substantial deficiencies in standardization and quality control within current organoid culture systems. For instance, the lack of unified standards across laboratories regarding sample pretreatment, medium formulations, matrix selection, and culture conditions may lead to significant fluctuations in success rates for the same sample type across different studies. Furthermore, tumor purity, cell viability, and the presence of normal cells or matrix contamination are critical factors affecting organoid formation and biological representativeness. Establishing unified sample evaluation standards, optimizing culture systems, and validating organoid-tumor consistency through multi-omics technologies represent key directions for enhancing organoid model reproducibility and clinical translational value. Therefore, advancing the clinical application of LCO technology requires not only further optimization of culture strategies for various sample types but also strengthened methodological standardization and quality control systems. This will ensure organoid models reliably and stably reproduce the biological characteristics of patient tumors, laying a solid foundation for their use in personalized drug screening and precision medicine.

**Table 2:** Comparison of success rates for the establishment of LCO models under different sample sources and culture conditions.

Research	Sample source	Cultivation system	Matrix materials	Breeding method	Success rate
Kim M, et al., 2019 (36)	Surgical resection specimen	RPMI 1640 + Growth factor(EGF10, FGF4, FGF7, Noggin, Wnt3a)	Collagen I	Traditional 3D suspension culture	~87%
Han Y. 2021 (37)	Surgical or biopsy tissue	Advanced DMEM/F12 + Growth factor(EGF Noggin R-Spondin 1)	Matrigel	Traditional 3D suspension culture	~89%
Wang HM, et al., 2023 (38)	Surgical resection specimen, malignant effusion specimen	Advanced DMEM/F12 + Growth factor(EGF, FGF10, HGF)	Matrigel	Traditional 3D suspension culture	~76%
Zhang Z, et al., 2014 (39)	Patient peripheral blood sample	/	Collagen I and Matrigel	Microfluid chip culture	~74%
Hu Y, et al., 2021 (40)	Surgical resection specimen	DMEM/F12 + Growth factor(FGF7, FGF10, R-Spondin, Noggin15, 25, 26)	Matrigel	Microfluid chip culture	~80%
Kim H, et al., 2022 (41)	Surgical resection specimen	NBA, GIBCO + Growth factor(EGF R&D, et al)	Collagen I	Microfluid chip culture	~80%

Success rate, % of samples that formed organoids.

### 2.3 Unique advantages of organoid technology in lung cancer research

In the research realm of preclinical lung cancer models, common types include cancer cell line (CCL) culture model, primary tumor cell (PTC) culture model, patient-derived xenograft (PDX) model, cell line-derived xenograft (CDX) tumor model, genetically engineered mouse model (GEMM), and LCO model (42). Compared to traditional tumor models, LCO offers several unique advantages in multiple aspects. It can highly simulate tumor tissue architecture, cellular heterogeneity, molecular characteristics, and the tumor microenvironment in an *in vitro* three-dimensional culture system, accurately reproducing the pathophysiological processes of lung cancer initiation, progression, and drug resistance, effectively bridging the gap between genotype and phenotype (43). Additionally, it features a shorter cultivation cycle, higher throughput, the capability for gene editing, and suitability for personalized drug screening, making it a powerful tool for lung cancer research (**Table 3**). However, the emergence of organoid models is not intended to replace traditional research models. Integrating organoids with conventional models holds promise for leveraging the complementary strengths of each approach, thereby enhancing the clinical predictive value and translational efficiency of lung cancer research. For instance, organoids can be employed for initial drug screening, with results validated *in vivo* using PDX or GEMM models. They can also be combined with CCL to investigate the functions of specific genes in environments more closely resembling physiological three-dimensional conditions.

**Table 3:** Comparison of advantages and disadvantages of common preclinical lung cancer models.

Model type	Advantage	Limitation
Cancer cell line	A large number of tumor cell populations can be obtained Selection may be based on phenotype or genotype	Tumor cell lines may have undergone multiple passages and selection, and do not completely retain the characteristics of primary tumors Not have human immune microenvironment
Primary tumor	Closer to the tumor cells *in vivo* and retains some biological characteristics of the primary tumor Having abundant specimen sources	Not have human immune microenvironment Lacking intercellular interactions Being easily disturbed by external factors, such as bacterial and fungal contamination
Patient-derived xenograft	Preserving the heterogeneity and complexity of the original tumor	The construction cost is high Cancerous tissues or cells of the patient are not easily obtained The time of tumor formation is long and the rate of tumor formation is low Lacking patient’s natural tumor microenvironment Exhibiting non-species-specific interaction and genetic drift between tumor and host
Cell line-derived xenograft	The xenograft tumor model established by inoculating human-derived tumor cell lines into immunodeficient mice grows rapidly The procedure is relatively simple and can be used in cohorts of animals bearing synchronous tumors for drug intervention studies Evaluating the development and progression of tumors	Low intra-tumoral heterogeneity, incomplete retention of the biological characteristics of the primary tumor Usually clonal and homogeneous, unable to fully mimic the complexity and diversity of primary tumors Not have human immune microenvironment
Genetically engineered mouse model	Can specify the initiating oncogenic lesions and the cells of origin of the tumor, which belong to *in situ* tumors Possessing complete immune system	Modeling time is difficult to master and the cost is high Low uptake rate of transgenes may affect the efficiency of model construction Not have human immune microenvironment
LCO	Highly similar to the original tumor tissue in terms of tissue structure, genome, transcriptome, and function, it can well concentrate the main of the original tumor Imitating the microenvironment of the original tumor Timely, strong proliferation ability, high stability, good manipulability High success rate in cultivation Can be used for high-throughput screening Can be cryopreserved for use as a biobank	Complex construction process, high technical difficulty, and high investment costs Limited cell development and low cell maturity Different tumors have very complex TMEs, and current organoid technology cannot fully and accurately replicate the patient’s specific immune environment The organoids currently being developed lack vascular structures, making it difficult for nutrients and oxygen to reach the core of the organoids, and unable to remove metabolic waste in time, thus limiting the growth rate and size of LCO, and even causing extensive cell death in the later of culture

TME, tumor micro environment; LCO, lung cancer organoid.

## 3 Exploration of the mechanism of lung cancer occurrence and metastasis based on organoid technology

The complexity of lung cancer is primarily manifested in the diversity of its etiologies, mechanisms, and metastatic pathways. Multiple risk factors, such as smoking, air pollution, occupational exposure, and ionizing radiation, can all contribute to the development of lung cancer (44). Studies have shown that lung lesions can be divided into three characteristic pathways, including signal transduction pathways that stimulate cell growth, pathways related to tumor suppressor genes, and pathways related to cell evasion of apoptosis. These three pathways can steer cells towards a malignant phenotype, subsequently inducing lung cancer (45). Lymphatic metastasis, hematogenous metastasis, direct diffusion, and intrabronchial dissemination can transport primary lung tumors to other tissues and organs in the body, forming metastatic tumors. Furthermore, lung cancer exhibits high heterogeneity, meaning that tumors in different patients or in different parts of the same patient’s body differ in gene expression, molecular markers, growth rate, and response to treatment. These unique characteristics make precision treatment for lung cancer particularly challenging. The emergence of organoid technology provides a novel approach and method for exploring the mechanisms of lung cancer occurrence and metastasis, paving a new path for the development of precision medicine for lung cancer.

### 3.1 Simulate the dynamic process of lung cancer occurrence and development

The development of tumors is a dynamically changing process, and its mutations often originate from rapid DNA replication in tumor cells, including chromosomal instability, microsatellite instability, and changes in epigenetic modifications (46). Given the diversity of tumor etiologies and the complexity of regulatory mechanisms, exploring its pathogenesis requires a multi-pronged approach, especially at the molecular level, such as tumor gene regulation and signal transduction (47). Studies based on LCOs have successively demonstrated the critical role of β-catenin and the Wnt signaling pathway in maintaining the stability of lung progenitor cell proliferation (48), as well as the influence of the *SRY-like HMG box 2 (SOX2)* gene and Notch pathway on the development of type 2 respiratory epithelial adenocarcinoma caused by *KRAS* gene mutations (49). In NSCLC, common mutated genes include *EGFR*, *ALK*, and *KRAS* (50), as well as tumor suppressor genes *TP53*, *KEAP1*, *STK11*, and *NF1* (51, 52). Compared to other gene mutations, the frequency of *KRAS* mutations remains almost constant across different tumor progression stages (6, 53), suggesting its important role in the initiation of tumors (54). Feng Jianqi et al. (54) successfully constructed a heterozygous human embryonic stem cell line with endogenous *KRASG12D/+* mutations through gene editing, and the cells maintained pluripotency despite having a normal karyotype. The experiment differentiated the constructed mutant cells into human alveolar organoids *in vitro* and found significant differences in the morphological structure between wild-type human embryonic stem cells (WT) and *KRASG12D/+* mutant human embryonic stem cells differentiated into alveolar organoids. Compared to WT, organoids carrying the *KRASG12D/+* mutation exhibited the following distinct characteristics: ①accelerated cell proliferation, increased number of apoptotic and dead cells; ②downregulation of differentiation and maturation markers, upregulation of development-related markers, and cells tending to maintain a progenitor state; ③significant changes in the expression of extracellular matrix-related genes, abnormal activation of tumor-related signaling pathways, and upregulation of cancer tissue expression. These are early characteristics of tumor development. Thus, it can be seen that the differentiation of stem cells into organoids *in vitro* can simulate and reproduce the early characteristics of cancer development, also confirming the hypothesis that *KRASG12D/+* mutations can induce tumor formation. Transferring organoids exhibiting differential phenotypes *in vitro* into the subrenal capsule of immunodeficient mice provides a new platform for studying tumor progression (54). Mice possess a complex microenvironment necessary for tumor growth, which is conducive to tumor formation and development. However, to enable organoids to simulate more malignant lung tumors, mutations in a single gene are insufficient to meet this requirement. Combining mutations in more oncogenic genes and the inactivation of tumor suppressor genes may yield a wider range of lung cancer models. Researchers believe that the use of organoid systems for *in vitro* manipulation and subsequent transplantation can make a significant contribution to exploring the pathogenesis and progression of lung cancer.

### 3.2 Organoid study on heterogeneity and drug resistance of lung cancer cells

The heterogeneity and drug resistance of lung cancer cells pose significant challenges to the precision treatment of lung cancer to some extent. Previous explorations on lung cancer were mainly based on established tumor cell lines and patient-derived xenograft models, but these traditional models still exhibit some limitations in practical applications, such as the inability to fully maintain all the genetic characteristics of the original tumor *in situ* and the lack of individual heterogeneity (55). As an emerging research tool, organoids, once successfully constructed, exhibit high similarity to the original tumor tissue in terms of tissue structure, genome, transcriptome, and function (36), accurately preserving the heterogeneity of the patient’s tumor (46). This complements the gaps and deficiencies in traditional models and provides tremendous clinical guidance value for decision-making and personalized treatment of anti-tumor drugs for lung cancer.

With the advent of epidermal growth factor receptor tyrosine kinase inhibitors (EGFR-TKIs), the field of precision medicine has ushered in a new chapter. The abnormal activation of the PI3K/AKT/mTOR pathway (referred to as the PI3K pathway) serves as a significant mechanism of EGFR-TKI resistance in lung cancer, representing a bottleneck issue in current targeted therapy for lung cancer (56). In order to explore methods for reversing such resistance with traditional Chinese medicine, Zhu Yanjuan et al. (56) employed 3D culture methods to cultivate PC-9-PIK3CA-M cell clusters (*EGFR/PIK3CA* double mutation), H1650 cell clusters (*EGFR* mutation combined with *PTEN* deletion), and an *EGFR/PIK3CA* double mutation LCO. These served as models of EGFR-TKI resistance caused by abnormal activation of the PI3K pathway, exploring the mechanism by which heat-clearing traditional Chinese medicine reverses gefitinib resistance. This experiment not only introduced organoid technology into the field of traditional Chinese medicine research, achieving a significant breakthrough at the technical level, but also enriched the theory of traditional Chinese medicine in treating lung cancer. It provided valuable insights for screening effective prescriptions to reverse EGFR-TKI resistance in lung cancer caused by abnormal activation of the PI3K pathway.

### 3.3 Organoid simulation and analysis of lung cancer metastasis mechanism

The recurrence and metastasis of lung cancer are primarily driven by a small number of tumor cells with high aggressiveness and metastatic potential. These cells can penetrate the basement membrane at the early stage of the disease, enter the blood circulation, and disseminate to distant sites as CTCs (57). Studies have shown that these CTCs play a crucial role in the recurrence and metastasis of lung cancer. However, their scarcity and potential biological differences pose significant challenges to the study of their functions. In recent years, 3D culture technology has developed rapidly, and the establishment of organoid models based on CTCs has provided a new research platform for exploring the biogenetic information related to CTCs and the mechanisms of tumor invasion and metastasis (58). Yuan Ligong (57), Li Guoliang (58), and others have established a 3D *in vitro* LCO culture system using CTCs derived from patients’ pulmonary venous blood. This system has verified the existence of different subpopulations of cells within CTCs and the heterogeneity of tumor cell populations. Notably, the presence of highly motile and epithelial phenotypic cell populations may be associated with cell proliferation and metastasis. Furthermore, they have discovered that the presence of neutrophil extracellular traps (NETs) is also a significant force in tumor formation and migration.

**Figure 2:**
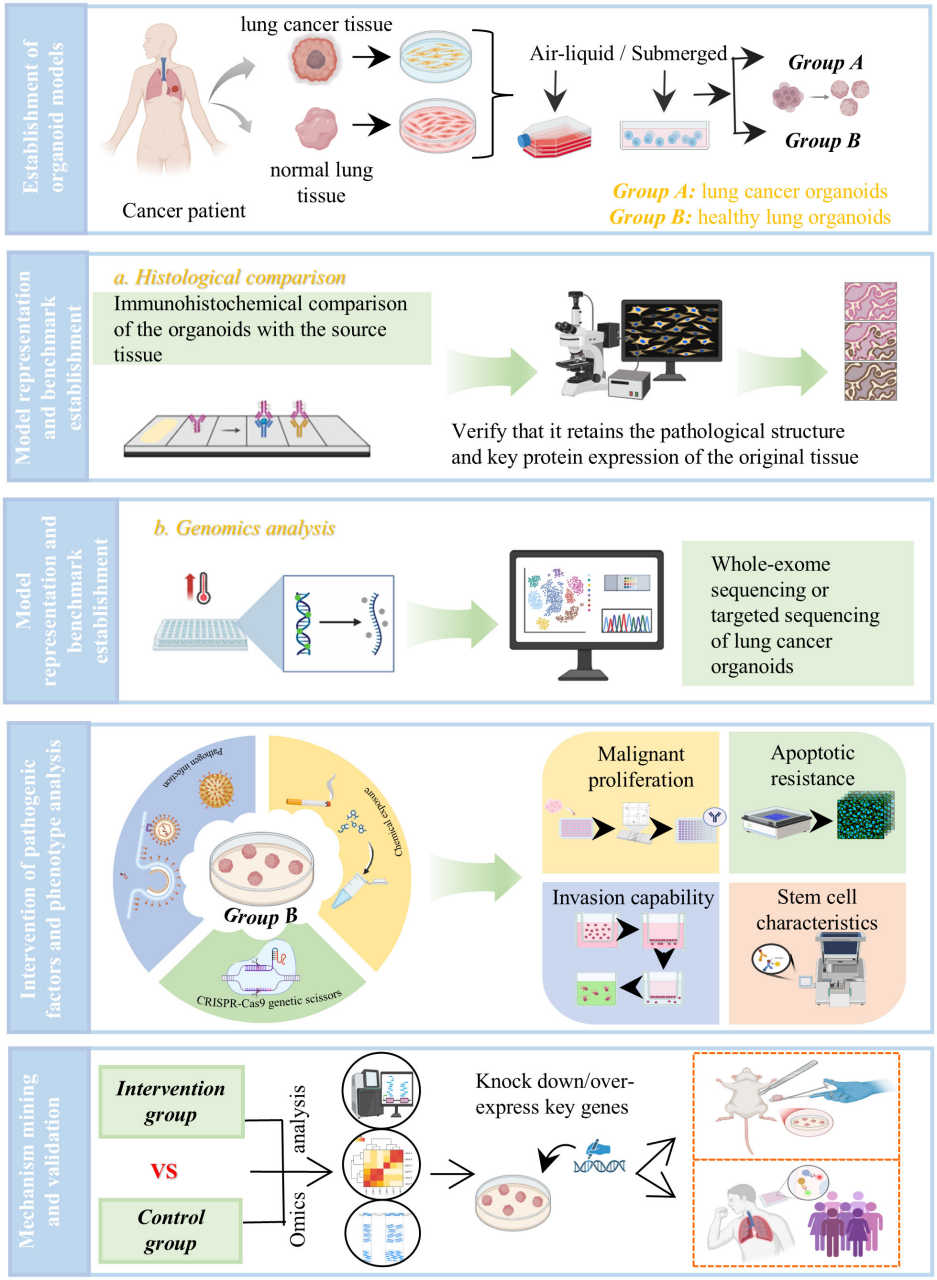
A research strategy for investigating the pathogenesis of lung cancer using LCO. To investigate the mechanism of lung cancer in depth, we can use organoids for simulation experiments. The experimental procedure mainly includes the following five key steps: establishment of organoid models, model representation and benchmark establishment, intervention of pathogenic factors and phenotype analysis, mechanism mining, *in vivo* and *in vitro* validation. **(A)** This study first established LCOs and normal lung organoids (NLOs) from patient-derived paired lung cancer and adjacent normal tissues. **(B)** These models then underwent rigorous quality control through histopathological, genomic, and functional assessments. **(C)** Carcinogenic processes are modeled by applying genetic (e. g., CRISPR-Cas9-mediated oncogenic mutation), chemical (e. g., cigarette smoke extract exposure), or pathogen-based interventions to NLDOs, with LCOs serving as native disease benchmarks. **(D)** Malignant transformation phenotypes are screened through multiplexed analyses, and differentially expressed genes and pathways are identified using multi-omics approaches including transcriptomics and epigenomics. **(E)** Finally, key mechanisms mediated by candidate genes or signaling pathways in lung tumorigenesis are validated at multiple levels through functional rescue experiments, *in vivo* xenograft models, and clinical cohort correlation, providing a mechanistic basis for targeted intervention.

Liu Wenwen (59) constructed a microfluidic bionic chip model for lung cancer brain metastasis and investigated its molecular mechanisms. Based on microfluidic chip-based organoid construction technology, she applied a bionic blood-brain barrier structure to a multi-organ microfluidic chip for lung cancer brain metastasis, reproducing the entire pathological process of lung cancer brain metastasis and achieving dynamic visualization and monitoring of the metastatic trajectory of lung cancer cells. This provided a reliable new methodological platform for exploring the molecular mechanisms related to blood-brain barrier disruption in lung adenocarcinoma brain metastasis. Yao Yuanshan et al. (60) demonstrated that the TNF receptor superfamily member herpesvirus entry mediator (HVEM-Fc) can activate the immune system against lung cancer *in vivo* and in organoid models. (**Figure 2**).

## 4 Applications of LCOs in drug screening and efficacy prediction

### 4.1 Drug screening

**Figure 3:**
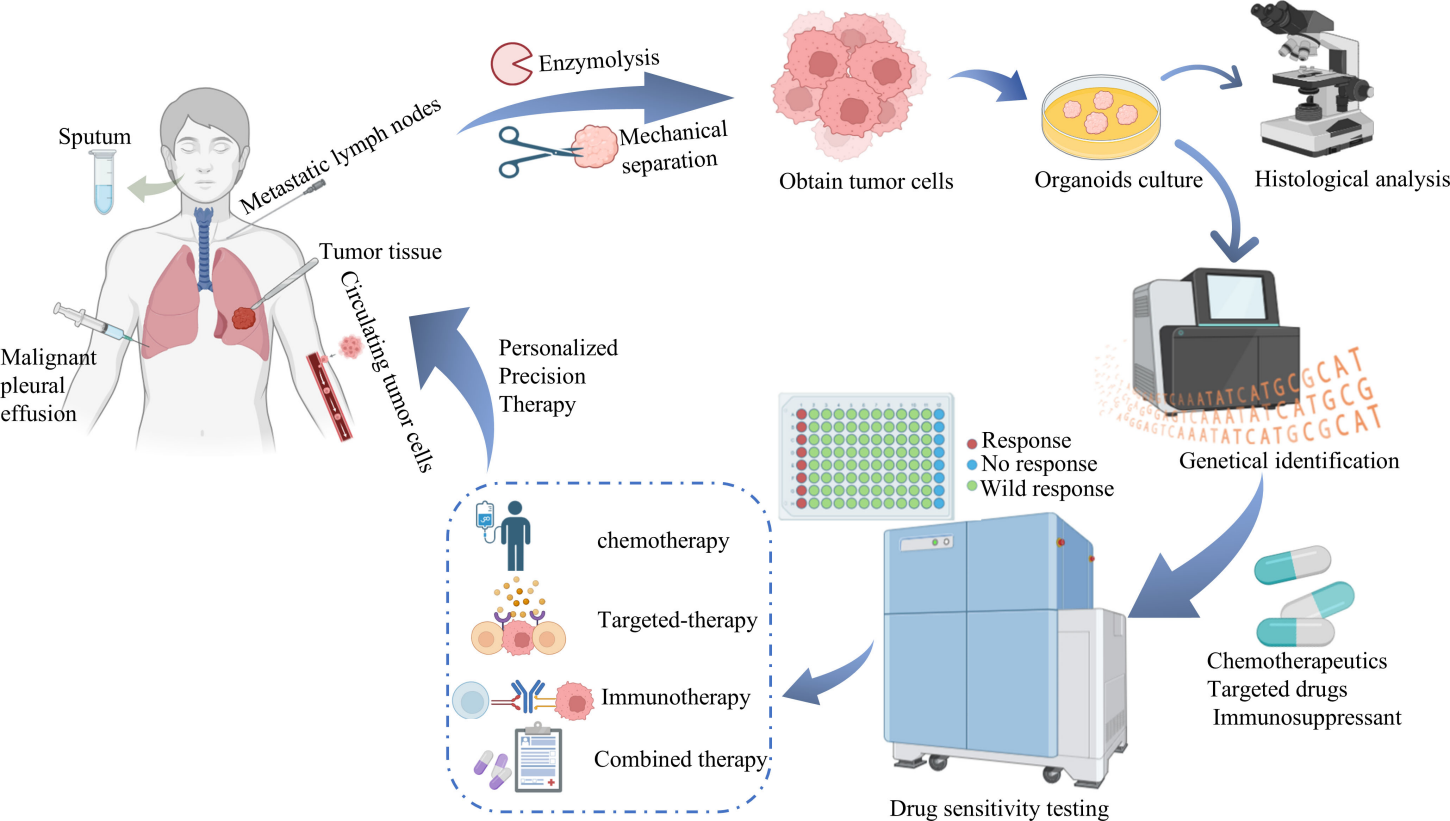
Establishment and validation of PDLCO and its application in precision medicine. All kinds of tissues (sputum, surgically resected tissues, metastatic lymph nodes, pleural effusion, and circulating tumor cells) were obtained from patients’ bodies, and lung cancer tumor cells were isolated and embedded in hydrogel for culture. PDLCO was verified through pathological analysis and gene identification. The cultured PDLCO can be directly used for drug screening of a variety of commonly used first-line and second-line drugs, including chemotherapy drugs, immunotherapy, etc. Based on the results of drug screening and the characteristics of patients, the most suitable sensitive drugs are selected, and personalized treatment schemes are formulated to achieve the goal of precision medicine.

LCOs can simulate the tumor characteristics of different patients, which provides a reliable *in vitro* model for the screening and efficacy assessment of targeted drugs. By comparing the sensitivity of different targeted drugs to LCOs, researchers can screen the most effective combination of targeted drugs and provide individualized treatment plans for patients (61). In 2019, In 2019, Minsuh et al. (36) successfully cultivated LCOs and normal bronchial organoids, from the tissues of lung cancer patients. This study investigated the correlation between the response of LCOs to chemotherapeutic drugs and their genetic mutations, which lays a solid foundation for the future development of targeted therapies. In 2020, Shi et al. (62) utilized surgically resected NSCLC patient tissues and the previously established PDX model, successfully cultured and established NSCLC organoids. The results showed that NSCLC organoids not only accurately reflected the histological characteristics of the patient and PDX tumors but also retained tumorigenicity and sensitivity to targeted therapeutic agents consistent with the parental tumors. Therefore, NSCLC organoids can be used as an effective tool for validation or discovery of biomarker-drug combinations. In recent years, LCOs have also been combined with other technologies to achieve high-throughput drug screening. Zhu (63) led a team to construct a dynamic microphysiological system chip platform (MSCP) using spheroids and organoid to develop a high-throughput lung cancer sphere model for the simultaneous evaluation of four chemotherapeutic agents (cisplatin, docetaxel, pemetrexed, and adriamycin) simultaneously to validate their efficacy. The results showed that MSCP, with its ability to rapidly construct high-throughput microphysiological systems and evaluate multiple drugs simultaneously, is a powerful platform for studying microphysiological systems, and has broad application prospects in personalized medicine, drug development and disease modeling. Organoid technology continues to advance, providing new technical means for large-scale drug screening (**Figure 3**).

However, personalized drug screening using LCOs also faces inherent challenges, with its core limitation being excessive individualization: models and screening results developed for one patient are often not directly applicable to others. This significantly restricts its efficiency as a universal therapeutic platform (64). To overcome this challenge and control costs, future personalized drug screening strategies could adopt the following approach: First, classify LCOs into specific molecular subtypes (e.g., EGFR-mutant, ALK-fusion) based on their genomic and transcriptomic sequencing results. Subsequently, conduct precise high-throughput screening using known effective drugs and candidate drug libraries for that subtype. This approach significantly reduces unnecessary testing and lowers costs. Furthermore, the standardization and scaling of organoid culture, automated drug delivery, and phenotypic analysis technologies are crucial for decreasing the screening cost per model. Microfluidic platforms (65) represent a major advancement in this direction, enabling high-throughput, low-reagent-consumption parallel testing through miniaturization and integration.

### 4.2 Efficacy prediction

LCOs are highly analog, personalized, efficient and instructive, and play an incomparable role in efficacy prediction. Organoids have shown excellent efficacy prediction in a variety of tumors, and much progress has been made in the drug research of lung cancer. Based on the patient-derived organoid (PDO) technique, Lee et al. (66) proposed a multiparametric analysis method-cancer organoid-based diagnosis reactivity prediction (CODRP). They tested PDOs from eight lung cancer patients to validate the CODRP index, categorized the responses to three ALK-targeted drugs (crizotinib, alectinib, and buxtitinib) according to ALK rearrangement status, and validated the results to be consistent with the clinical response to the drug therapy. This finding suggests that LCOs can be used for drug efficacy prediction, providing important reference value for physicians to develop precise treatment plans. The same conclusion was also seen in the experiment of Deben et al. (67), who combined Auranofin (AF) with 11 drugs based on their drug screening results in NSCLC-derived carcinoid organoids, pancreatic ductal adenocarcinoma (PDAC), and non-cancerous lung carcinoid organs. Comparative transcriptomic analysis across the three organoid types revealed that at clinically relevant concentrations (<1 μM), AF specifically upregulated cell death-related gene pathways in cancer cells while minimally affecting normal cells, molecularly confirming its preferential cytotoxic action. Furthermore, by correlating gene expression data with drug responses, they identified low levels of CA12 as an effective predictor of AF sensitivity, offering potential pathways for combination therapy and personalized application of AF. On the other hand, based on the results of predicting the efficacy of lung cancer carcinoids, it is also possible to change drugs and adjust or modify the treatment regimen in time to avoid ineffective treatment. In a study conducted Wang et al. (38), a patient was first treated with first-line ectinib due to *EGFR* exon 19 deletion, and the efficacy assessment showed localized progression. Subsequently, based on the results of a drug sensitivity trial in LCOs, the patient was switched to treatment with ositinib and achieved sustained local remission. Therefore, in the future, LCOs can also be applied to efficacy monitoring, which can help maximize efficacy and durability during treatment by monitoring changes in the model in real time and adjusting the treatment regimen in a timely manner as needed, thus achieving more precise treatment.

## 5 Integration of LCO with emerging technologies

Synergistic integration of tumor organoid with other technologies can help overcome their limitations and refine them into more adaptable model systems that encapsulate the complex stroma of cancer, inter- and intra-organ communication, and potential for multi-organ metastasis. A detailed description of the three emerging technologies related to LCOs, as well as a separate summary of their respective strengths and weaknesses, is shown in **Table 4**.

**Table 4:** Comparison of advantages and disadvantages of three emerging technologies applied to organoids.

Technical type	Advantage	Disadvantage
Organs-on-Chips	Accurately simulate the function of a single organ Provide a controllable and dynamic microenvironment Ethical advantages	The construction of technology and evaluation system has not yet formed a unified standard It is difficult to obtain results that are completely consistent with the pathological and physiological reactions of the human body for simple splicing Most of the fluxes are low
Three-dimensional printing	Beneficial for constructing complex functional vascular networks of organoids Provide opportunities for organ donation scarcity	High cost and time-consuming It is difficult to fully simulate the actual composition of official specific ECM
CRISPR-Cas9	Fast and efficient Accurately edit or label specific genes in organoids To construct an ideal organoid model	Need to consider the specificity of various types of cells Usually requires a large number of organoids There may be gene omissions Involving ethical and legal issues

ECM, extracellular matrix.

### 5.1 Organs-on-Chips

Organs-on-Chips (OoCs) are organ-physiological microsystems constructed on a chip. By integrating organoid models with advanced microfluidic and tissue-engineering techniques (68), OoCs can closely mimic the structure, function, and microenvironment of human organs. Microfluidic technology is a technique for processing and manipulating minute volumes of fluid at the microscopic scale. It enables the precise regulation of oxygen concentration, mechanical stress, and chemical drug concentrations to elucidate the tumor microenvironment relevant to tumor progression, metastasis, and drug metabolism. This technology has played a pivotal role in establishing organoid research models (65). In recent years, organoid-on-chip systems, particularly organoid microarrays, have emerged as a promising technology for clinical lung cancer research. For example, Hu et al. (40) processed lung tumor tissues into fragments, expanded them into LCOs within three days, and then cultured the LCOs on an integrated superhydrophobic microporous array chip (InSMAR-chip) for high-throughput drug screening. The drug-response profiles obtained from the chip-based LCOs showed strong consistency with the clinical outcomes observed in the original patients, demonstrating that the combination of LCOs with micro-engineered chip platforms is both feasible and clinically relevant for predicting patient-specific therapeutic responses.

Despite the immense potential of OoCs as an emerging technology, they still face multiple challenges. The first problem to be solved is that the construction method and evaluation system of OoCs technology have not reached a unified standard, posing significant obstacles to its transition from preclinical research to clinical practice (69). Secondly, most OoCs applications are currently limited by low throughput. In the early stage of drug research and development, high-throughput platforms play a key role in reducing research and development costs. However, the throughput level of most organ chip platforms is relatively low, which limits the wide application of mature organ chips in the field of drug screening. Finally, the response of the current organ chip platform is mostly limited to the isolated state, rather than the overall system response. While organ chips can accurately simulate the functions of specific organs to some extent, they are still in the primary stage of simple combination, and the system integration is not high. Therefore, whether it is a single organ chip or a combination of multiple organ chips, it is difficult to obtain results that are completely consistent with human pathophysiological responses (70).

### 5.2 Three-dimensional printing

Tumor-like organoids have great potential in reconstructing three-dimensional structures and heterogeneous cellular components. However, their ability to simulate other key elements such as TME is limited, including tumor-specific biochemical/biophysical properties, anatomical scale, graded blood/lymphatic vessels, and fluid dynamics (71). Combining 3D bioprinting technology with tumor-like organoids can overcome these limitations and create a more comprehensive, accurate, and clinically relevant model. 3D printing technology can be defined as a medical manufacturing technique that “transforms” digital images into 3D solid objects by continuously printing thin-layer materials (72). In the three-dimensional bioprinting of organoids, the precise deposition of cells, biomaterials, and sacrificial inks makes it possible to manufacture organoids with predetermined vascular channels, thereby enabling more precise control over vascular structures. However, the potential of this technology goes far beyond simple vascularization. It also provides the possibility to solve the organ donation crisis by eventually developing customized and transplantable organs such as kidneys (73) and livers (74). However, challenges still exist - further research and optimization are needed to fully replicate the hierarchical complexity of natural blood vessels and ensure the long-term function of grafts (75). Secondly, in personalized medicine, building a three-dimensional tumor model for each patient may be expensive and time-consuming. This sometimes exceeds the time limit available for making large bioprinted tissues. Especially when it is necessary to switch between different materials or achieve very high bioprinting resolution, the time required to manufacture functional structures increases significantly. In addition, although biological inks for 3D printing can induce some organ-like behaviors in cells, they usually still represent a “synthetic” environment, and it is difficult to fully simulate the actual composition of organ-specific ECM (76).

At present, 3D printing has been applied to tumor organ models such as hepatocellular carcinoma (77), cervical cancer (78), colorectal cancer (79), and lung cancer (80). These organoids exhibit the structure and function of real organs, which is expected to promote bioprinting to play a huge role in drug screening, disease simulation, and new technology research. In the future, we also anticipate more integration of 3D printing technology with LCOs, thereby offering new insights and approaches for lung cancer research and treatment.

### 5.3 CRISPR-Cas9

CRISPR/Cas9 is a gene editing tool based on the immune system of bacteria and archaea. It consists of clustered regularly interspaced short palindromic repeats (CRISPR) and CRISPR-associated protein 9 (Cas9) (81). CRISPR/Cas9 has emerged as an effective method for altering the genomes of various organisms, and is widely used for gene editing and activating or inhibiting gene expression (82). Therefore, CRISPR/Cas9 is expected to provide an effective method to analyze the mechanism of tumor occurrence, determine the target of drug development, and introduce gene mutations of specific diseases into tumor-like organs, thereby establishing disease models and accelerating cancer research. Currently, CRISPR-Cas9 technology has been applied in LCOs. Li et al. (83) used gene editing technology to inactivate *sex-determining region Y-box transcription factor 9* (SOX9) in human embryonic stem cells, and then induced these cells to differentiate into lung-like organs to study the role of SOX9 in the development of human lung epithelium. Hai (84) utilized CRISPR gene editing technology to delete multiple tumor suppressor genes from mouse lung-like organs that express Cre-dependent *sex-determining region Y-box transcription factor 2* (SOX2). They explored the therapeutic and immune effects of combining *programmed death-1* (PD-1) blockade with WEE1 inhibition in both mouse organoid models and cell lines derived from patients with lung squamous cell carcinoma (LSCC). Their research demonstrated that employing the CRISPR-Cas9 gene editing system for multiple gene edits in mouse lung-like organs is an efficient and rapid approach, capable of generating a lung squamous cell carcinoma model that closely mimics human disease at both the genomic and phenotypic levels. A et al. (85) employed CRISPR/Cas9 gene editing to silence *RBMS3* in LCOs and discovered that the loss of *RBMS3* synergizes with *BRAFV600E* to trigger lung tumor formation. This revelation offers deeper insights into the molecular mechanisms underlying mutant BRAF-driven lung cancer and unveils potentially more effective treatment strategies for this disease.

CRISPR-Cas9 technology has become a hotspot in animal modeling research in recent years because of its high specificity, high efficiency, versatility and other advantages (86, 87), but there are still some problems to be solved in the combination of CRISPR-Cas9 and organoid technology. First of all, the advantage of directed differentiation of organoids over traditional stem cells is that they can fully consider the complex interactions between different cell populations, so as to more comprehensively simulate the microenvironment of organs. Therefore, when CRISPR-Cas9 is used for organoid screening research, cell specificity becomes a factor that must be considered, which is directly related to the accuracy of the final screening results (88); Secondly, the use of CRISPR-Cas9 technology usually requires a large number of organoids, which has high requirements for the materials and costs of the experiment; In addition, when certain genomes are selected for research, gene omission is inevitable, which makes it impossible to comprehensively study and understand the functions of the whole genome; The ethical and legal issues facing the use of CRISPR-Cas9 technology also deserve our consideration.

## 6 Challenges and future perspectives

LCOs are now widely employed in both basic and translational lung cancer research. However, certain limitations currently hinder their potential use in personalized medicine and drug development. The incomplete reproduction of the tumor microenvironment, absence of systemic pharmacokinetics and dynamic physiological processes, inherent model instability and representational bias, along with barriers to clinical translation, poses obstacles to utilizing organoids for replicating authentic microenvironments and drug responses.

### 6.1 Limitations and challenges

First, the most fundamental limitation of LCOs lies in their inadequate representation of complex biological processes and dynamic systems within the body. This deficiency manifests in two key aspects: first, the tumor microenvironment is highly simplified. Most LCOs primarily enrich tumor epithelial cells during culture, resulting in the loss of critical immune cells (e.g., T cells and macrophages), stromal cells (e.g., cancer-associated fibroblasts), and vascular networks present in primary tumors (89). This model not only limits applications in immunotherapy and anti-angiogenic therapy but may also introduce culture artifacts. Second, LCOs lack systems pharmacology and translational dynamics. Static culture systems cannot simulate *in vivo* drug absorption, distribution, metabolism, and excretion (ADME) processes, nor account for physiological barriers like the blood-brain barrier. More critically, these models fail to reproduce the core steps of cancer metastasis, specifically the complete “invasion-colonization cycle” that encompasses invasion, intravasation, circulation, extravasation, and colonization (90). This limitation makes it difficult to study metastatic organ specificity and underlying mechanisms.

Second, the model itself faces inherent challenges regarding stability and representativeness. During prolonged passage, subclones with growth advantages may be excessively selected, causing organoid genomes and phenotypes to progressively deviate from the original tumor, a phenomenon termed phenotype-genotype decoupling (91), which compromises long-term reliability. Additionally, establishment rates vary significantly across histological subtypes (e.g., LSCC, SCLC) or molecular subtypes, leading to biases that may cause research cohorts to fail in representing true lung cancer heterogeneity.

Furthermore, significant barriers exist in translating LCOs from the laboratory to the bedside. Technologically, the process from sample acquisition to drug testing typically takes weeks or months, directly conflicting with the urgent clinical decision-making window for late-stage patients. The technical complexity and high costs of model construction and high-throughput screening also limit routine clinical adoption. At the regulatory level, the field currently lacks standardized production processes and rigorous quality control systems. This absence of standardization is the primary cause of poor data reproducibility and difficulties in cross-laboratory comparisons, potentially leading to data misinterpretation, resource waste, and ethical concerns (92).

### 6.2 Conclusions and future prospects

In summary, compared to traditional 2D cell cultures and animal models, LCOs address many of their limitations, providing an economical, efficient, and highly representative *in vitro* culture system. Since LCOs can better maintain the biological characteristics and heterogeneity of parental tumors, they have been widely used in drug screening and have shown great potential for predicting drug sensitivity. This is highly valuable for advancing targeted drug development, designing personalized treatment plans, progressing precision medicine, and investigating lung cancer pathogenesis.

With the advent of precision medicine and rapid biotechnology development, LCO models hold great promise for future lung cancer treatment. Future research should focus on improving the quality, stability, and scalability of LCOs for large-scale application. Additionally, integrating other advanced technologies, such as organs-on-chips, 3D bioprinting, gene sequencing, and immunotherapy, with traditional organoid models will help build a more comprehensive precision treatment system. Exploring bioengineering technologies to develop real-time monitoring platforms for tumor organoids could also provide new solutions to the challenge of dynamically tracking LCO responses.
